# Pharmacokinetics and Tissue Residue Profiles of Enrofloxacin in Crucian Carp (*Carassius auratus gibelio*) Following Single and Multiple Oral Administration

**DOI:** 10.3389/fvets.2022.872828

**Published:** 2022-04-14

**Authors:** Qi Shan, Heqing Huang, Guangming Zheng, Yi Yin, Xinping Zhu, Lisha Ma, Hao Zhou, Wenping Xie, Lichun Li, Shugui Liu, Jingxin Wang

**Affiliations:** ^1^Key Laboratory of Recreational Fisheries Research, Ministry of Agriculture and Key Laboratory of Aquatic Animal Immune Technology of Guangdong Province, Pearl River Fisheries Research Institute, Chinese Academic of Fishery Science, Guangzhou, China; ^2^Key Laboratory of Tropical and Subtropical Fishery Resource Application and Cultivation, Ministry of Agriculture, Pearl River Fisheries Research Institute, Chinese Academic of Fishery Science, Guangzhou, China; ^3^Guangdong Provincial Engineering Research Center of Public Health Detection and Assessment, School of Public Health, Guangdong Pharmaceutical University, Guangzhou, China

**Keywords:** enrofloxacin, pharmacokinetics, crucian carp, ciprofloxacin, tissue distribution, withdrawal time

## Abstract

The pharmacokinetics, tissue distribution, and elimination of enrofloxacin (ENR) and its metabolite ciprofloxacin (CIP) were investigated to the crucian carp (*Carassius auratus gibelio*) after single (20 mg/kg b. w.) and multiple oral administration (20 mg/kg b.w. one time daily for 5 days) at 28°C. The concentrations of ENR and CIP in the plasma and tested tissues (muscle/skin, liver, and kidney) were detected simultaneously by high-performance liquid chromatography (HPLC), and the pharmacokinetic data were analyzed with a non-compartmental model using WinNonLin 6.1 PK software (Pharsight Corporation, Mountain View, CA, USA). The pharmacokinetic characteristics of ENR in crucian carp exhibited slow absorption, wide tissue distribution, and long elimination half-life. In the single-dose group, the peak concentrations (Cmax) of ENR in the plasma, muscle/skin, liver, and kidney were 8.93 μg/mL, 13.9 μg/g, 31.2 μg/g, and 27.3 μg/g, respectively, observed at 3 h, 6 h, 1 h, and 3 h after dosing. The elimination half-lives (T_1/2λ*z*_) of ENR in plasma, muscle/skin, liver, and kidney were calculated to be 67.4, 82.8, 94.4, and 114 h, respectively. In the multiple-dose group, the C_max_ of ENR in the plasma, muscle/skin, liver, and kidney were 18.4 μg/mL, 26.8 μg/g, 82.8 μg/g, and 74.5 μg/g, respectively, achieved at 3 h, 6 h, 1 h, and 1 h after the last dose. The T_1/2λ*z*_ of ENR in the plasma, muscle/skin, liver, and kidney were calculated to be 76.4 h, 91.5 h, 114 h, and 148 h, respectively. During the multiple-dose administration, significant accumulations of ENR and CIP were observed in the plasma and tissues of crucian carp, possibly due to their long elimination half-lives. In both dose groups, the AUC_0−∞_ for both ENR and CIP followed the order of liver > kidney > muscle/skin > plasma. The finding suggested that the liver may play an important role in the metabolism of ENR. According to the calculated PK/PD indices of C_max_/minimum inhibitory concentrations (MIC) and AUC_24h_/MIC, the multiple-dose regimen would be highly effective against pathogenic bacteria with a MIC value of ≤ 1.84 μg/ml. Depletion studies indicated that a withdrawal period of at least 29 or 32 days was necessary to guarantee food security after single or multiple oral gavage administration at 28°C.

## Introduction

Crucian carp (*Carassius auratus gibelio*), with the characteristics of fast growth, strong stress resistance, and high nutritional value, is an important freshwater fish in China (1). The annual production of crucian carp in 2020 exceeded 2.75 million tons. With the gradual development of intensive aquaculture, fish farming is facing many disease problems (2), such as *Rahnella aquatilis* (3), *Edwardsiella ictaluri* (4), *Aeromonas hydrophila* (5), which has caused huge economic losses to the breeding industry. Due to the lack of fish vaccines, antibiotic treatment has become a common way to fight bacterial infections.

Enrofloxacin (ENR) is a second-generation fluroquinolone developed for veterinary and aquaculture applications. It has a good bactericidal effect on most Gram-negative bacteria and some Gram-positive bacteria such as *Aeromonas hydrophila* (6), *Flavobacterium columnare* (7), *Yersinia ruckeri*, and *Vibrio anguillarum* (8, 9). Due to its excellent pharmacokinetic properties and broad-spectrum antibacterial activity, ENR is widely used to prevent and treat bacterial infections in aquatic animals. Quinolones pharmacokinetics have been studied in many animal species, such as Yellow River carp (10), sea bass (11), grass carp (12), turtles (13), Japanese quails (14), dogs (15), calves (16), pigs (17), and turkeys (18). However, almost all studies have focused on the pharmacokinetic characterization of single-dose administration in various animal species. There are very few studies on the pharmacokinetics of multiple-dose administration (19–21). For aquatic animals, antibacterial drugs are usually given multiple times in succession to achieve the desired therapeutic effect (22). Therefore, multi-dose pharmacokinetics has important clinical significance.

The present study aimed to investigate the pharmacokinetics, tissue distribution, and elimination of ENR and its metabolite ciprofloxacin (CIP) in crucian carp after single and multiple oral administration and further estimate the withdrawal periods of ENR in crucian carp administrated two regimens. This study provides crucial information for developing effective and reasonable enrofloxacin regimens and ensuring food security in crucian carp.

## Materials and Methods

### Chemical Reagents

The reference standards of ENR and CIP hydrochloride were supplied from Dr. Ehrenstorfer GmbH, Germany (Augsburg, Germany). The ENR powder for experimental use in crucian carp was donated by Guangzhou Xiande Biotechnology co., LTD (Guangzhou, China). Acetonitrile and n-hexane were HPLC-grade and acquired from J.T. Baker (Griesheim, Germany). The rest reagents used were analytical grade and obtained from Sigma-Aldrich Co. Ltd. (Shanghai, China).

### Animals

From a culture pond in Dongguan city of Guangdong province, 300 healthy crucian carp (mean body weight, 250 ± 20 g) with no history of drug use were obtained and divided into two groups, one for single-dose administration was further divided into 18 subgroups (*n* = 6), and the other for multiple-dose administration was further divided into 32 subgroups (*n* = 6). The fish in each subgroup were kept in a fiberglass tank (about 500 L) with constant aeration. The water temperature was maintained at 28°C with a heating rod. During the whole experiment, the pH of the water was 7.1–7.5, the ammonia concentration was <0.05 mg/L and the dissolved oxygen of the water was between 7.5 and 8.0 mg/L. Before the experiment, the tested fish were adapted to the environment in the tank for at least 2 weeks and fed with extruded feed two times a day. The fish were fasted for 1 day prior to the experiment and were not fed on the day of administration. During the course of the experiment, the fish were in good condition and no deaths occurred.

### Experimental Design

The method for the preparation of the ENR suspension is as follows: ENR was added into sterile water, and 1 M NaOH solution was added dropwise until dissolution was complete and the pH was about 10.5 and then mixed with blank feed (20%, w/v) to obtain a suspension with a final ENR concentration of 20 mg/mL. The suspension must be thoroughly mixed before each use. For oral administration, the ENR suspension was given to the fish by oral gavage using a 1-ml syringe attached to a 96-mm-long stainless-steel gavage needle. The fish in the single-dose group was administered once at a dose of 1 mL/kg b.w., while the fish in the multiple-dose group was administered at the same dose daily for 5 days. Following each dose administration, the treated fish were observed in a fresh tank for 5 min for possible regurgitation of ENR. The oral gavage was chosen rather than the most common route of medicated feed in order to administer an accurate dosage to the fish based on body weight, but the potential influence on the PK profile and residue depletion of enrofloxacin remains to be investigated. After drug administration, six fish of one subgroup were randomly sacrificed by a blow to the head and sampled at each time point. For the single-dose group, blood samples were collected before and at 5, 15, and 30 min; 1, 3, 6, 9, 12, and 24 h; and 3, 6, 10, 14, 20, 30, 40, and 60 days post-treatment, while tissue samples including the liver, kidney, and muscle plus skin were obtained at the same time except for the two time points of 5 and 15 min. For the multiple-dose group, the samples of blood, liver, kidney, and muscle plus skin were collected before and at 30 min; 1, 3, 6, 12, 24, 25, 27, 30, 48, 49, 51, 54, 72, 73, 75, 78, 96, 96.5, 97, 99, and 102 h; and the days of 5, 7, 10, 14, 18, 24, 34, 44, and 64 after the first dose, respectively. About 2 ml of blood samples were collected from the caudal vessel by using heparinized syringes, after centrifuging at 2,683 *g* for 10 min, the plasma was then transferred to another test tube. All samples were stored at −20°C for subsequent analysis.

### Analytical Method

The determination of ENR and CIP were carried out based on our previously published HPLC methods (19, 23). Shortly, 0.5 ml of plasma was extracted two times with 3 ml of acetonitrile. The acetonitrile extracts were combined into a 15 ml glass tube and blown to near dryness by nitrogen in a 40°C water bath. The dried samples were then dissolved in 0.5 ml of 20% acetonitrile water and filtered through a 0.22-μm nylon membrane for further analysis. For tissue samples, the homogenized tissue samples (2 g muscle/skin, 2 g liver, and 1 g kidney) were weighed, and 10 ml 1% acetic acid in acetonitrile was added. This mixture was vortexed for 2 min, sonicated for 20 min, and centrifuged for 10 min, the resulting supernatant was collected into a 50-ml glass tube and the extraction process was repeated. The combined extracts were concentrated under a flow of nitrogen to near dryness in a 40°C water bath and dissolved in 2 ml of 20% acetonitrile water (kidney was 1 ml), and then 3 ml of n-hexane was added to degrease. After centrifugation (7,000 *g* for 10 min), the lower liquid was filtered through a 0.22 μm nylon membrane for further analysis.

All analyses were carried out on a Waters e2695 Alliance HPLC system (Waters Corp., Milford, MA, USA) equipped with a Waters 2695 separation unit (an autosampler, an online degasser, a quaternary pump solvent management system, a gasket cleaning system, and a column heater), an Empower 2 chromatography data system, and a Waters 2475 multi λ fluorescence detector. Separation was achieved on a C18 column (Zorbax SB C-18, 4.6 × 250 mm, 5 μm; Agilent Technologies, Inc.) with a mobile phase that included ammonium phosphate buffer (pH 3.5; 10 mM) and acetonitrile (the ratio was 80:20, v: v). The injection volume was 20 μl, and the flow rate was 1.0 ml/min. The wavelengths of the excitation and emission were set at 280 nm and 460 nm, respectively. The limit of detection (LOD) was determined based on the concentration that produced the area of the signal 3 times that of the baseline noise; the limit of quantification (LOQ) was determined as the concentration that produced the ratio of the area of the signal to the baseline noise of 10. The accuracy of the method and system was verified by analyzing the different spiked samples (*n* = 5) three times on different days in the week, respectively. The levels of ENR and CIP spiked in the plasma and tissue samples (muscle/skin, liver, and kidney) were 0.03, 0.5, and 20 μg/ml (or μg/g), respectively. The extraction method was the same as the processing method of each tissue sample.

### Data Analysis

A non-compartmental model was used to analyze the concentration-time data of ENR and its metabolite CIP in crucian carp by WinNonlin 6.1 software(Pharsight Corporation, Mountain View, CA, USA). The PK parameters were calculated from average ENR or CIP concentration at each time point, such as peak time (T_max_), peak concentration (C_max_), terminal elimination half-life (T_1/2λ*z*_), area under the curve from time zero to infinity (AUC_0−∞_), area under the curve during a dose interval in steady state (AUC_ss_), concentration at the end of the dosing interval at steady state (Ct_rough_), average steady-state concentration (C_av_), degree fluctuation (DF), mean residence time (MRT), oral clearance (CL/F), and apparent volume of distribution (Vd/F). C_max_ and T_max_ were actual observed data and presented as mean values. AUC_0−∞_ was calculated by the linear trapezoidal rule and extrapolated to infinity. The extrapolated part of the AUC was always <10% of the total area. The T_1/2λ*z*_ was calculated as ln(2)/λz, where λz was the terminal elimination rate constant. The terminal half-life was accepted only when linear regression analysis was based on at least three time points in the elimination phase and the regression coefficient was (r^2^) >0.85. The PK analysis is usually performed only on the drug concentrations in plasma, but in this study, the PK analysis was performed on the drug concentrations in all the plasma and tested tissues in order to provide a more comprehensive comparison of the results among plasma and tissues. According to the regulations of China and the European Union (24, 25), the maximum total residues of ENR and CIP in fish muscle with adhering skin should be no more than 0.1 μg/g. The sum of concentrations of ENR and CIP from each fish at each time point was used to estimate the drug withdrawal time by using the application WT 1.4. According to EMA guidelines, the last seven sample times in the terminal elimination phase of muscle plus skin of crucian carp were used to calculate the drug withdrawal time (26). Using the statistical software package SPSS 19.0 (SPSS Inc., Chicago, IL, USA), an independent sample *t*-test was performed to check the differences between group means, and the *p* < 0.05 would be considered significant.

## Results

### Method Verification

**Figure 1:**
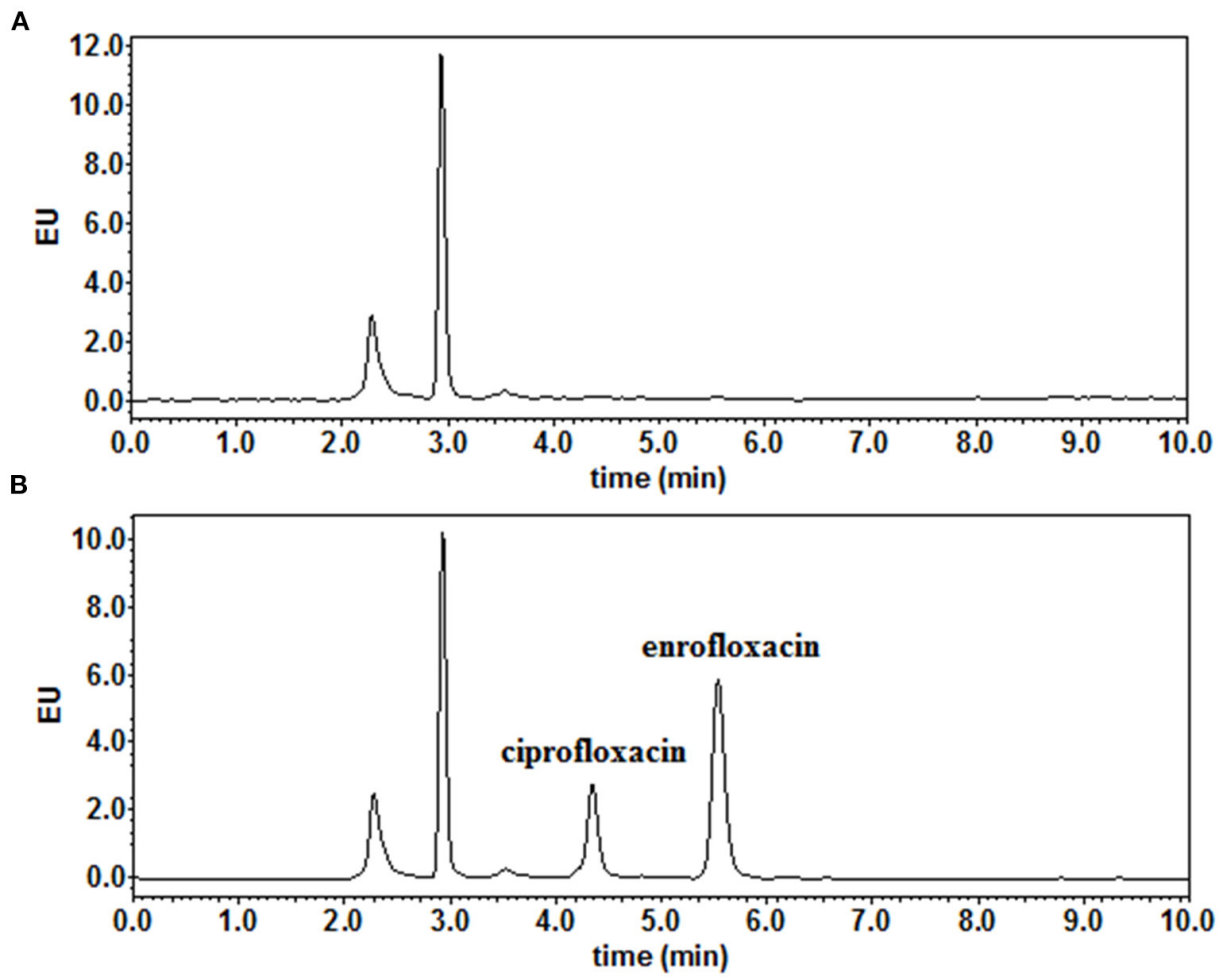
The representative chromatograms of **(A)** blank muscle/skin matrices of crucian carp and **(B)** muscle/skin samples spiked at 0.03 μg/g of enrofloxacin and ciprofloxacin.

High-performance liquid chromatography analysis method showed that the LOD and the LOQ for both ENR and CIP were 0.01 μg/ml (or μg/g) and 0.03 μg/ml (or μg/g), respectively (Figures 1A,B). In this experiment, the linear range of the standard curve was 0.03–20.0 μg/ml (or μg/g), and the correlation coefficients (R^2^) of all samples were higher than or equal to 0.9997. Samples with a concentration higher than the linear range were diluted with a blank matrix. The recovery rates of ENR and CIP in this experiment were 84.7–92.4 and 83.2–95.4%, respectively. The inter-assay and intra-assay coefficients of variation were both lower than 5.49 and 6.20% for ENR and CIP, respectively.

### Pharmacokinetics of ENR and CIP in the Single-Dose Oral Administration

**Figure 2:**
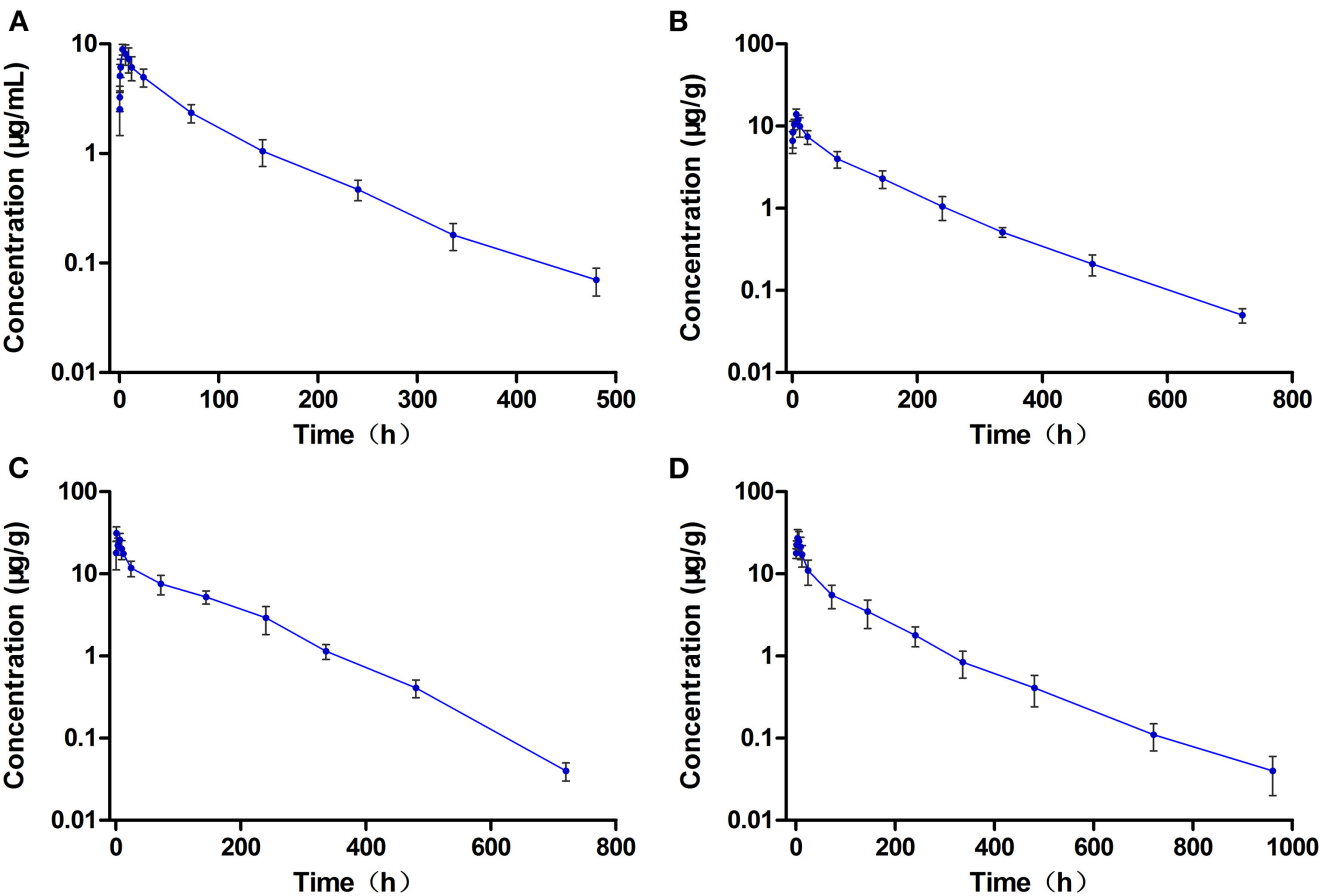
The concentration vs. time curves of enrofloxacin in the plasma **(A)**, muscle/skin **(B)**, liver **(C)**, and kidney **(D)** of crucian carp after a single oral dose of enrofloxacin (20 mg/kg b.w.) at 28°C (*n* = 6).

**Figure 3:**
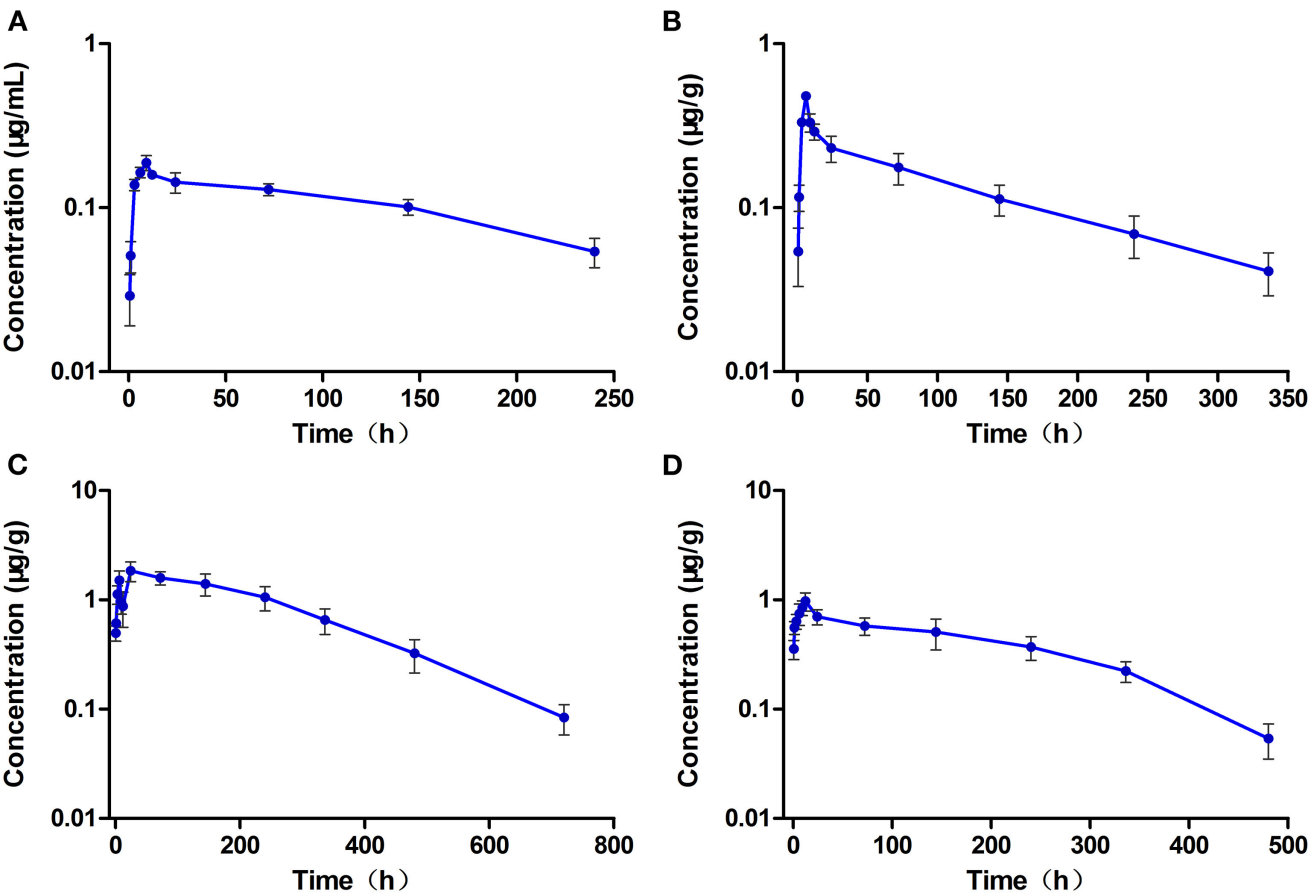
The concentration vs. time curves of ciprofloxacin in the plasma **(A)**, muscle/skin **(B)**, liver **(C)**, and kidney **(D)** of crucian carp after a single oral dose of enrofloxacin (20 mg/kg b.w.) at 28°C (*n* = 6).

The concentration-time curves of ENR and CIP in the plasma and tested tissues of crucian carp following single-dose oral administration are presented in Figures 2A–D, 3A–D, respectively. The peak concentrations (C_max_) of ENR were achieved at 3.0 h, 6.0 h, and 3.0 h in the plasma, muscle/skin, and kidney of crucian carp, respectively (Table 1). However, unlike in the plasma and tissues of muscle/skin and kidney, the C_max_ of ENR in the tissue of the liver was achieved at 1 h, then declined, and achieved the second peak concentration at 6.0 h. The highest C_max_ of 31.2 μg/g was observed in the liver, and for plasma, muscle/skin, and kidney, the C_max_ values were 8.93 μg/ml, 13.9 μg/g, and 27.3 μg/g, respectively. The values of T_1/2λ*z*_ in plasma, muscle/skin, liver, and kidney were 67.4 h, 82.8 h, 94.4 h, and 114 h independently, showing a slow elimination. The AUC_0−∞_ in the corresponding plasma and tissues was measured as 579 μg·h/ml, 1,059 μg·h/g, 2,131 μg·h/g, and 1,712 μg·h/g, respectively, and the plasma Vd/F was estimated as 3.36 L/kg, suggesting that ENR was distributed widely in crucian carp, and the ENR concentrations in the plasma were lower than those in the other tissues. The AUC_0−∞_ values of CIP in the plasma, muscle/skin, kidney, and liver were 10.5 μg.h/ml, 48.6 μg.h/g, 554 μg.h/g, and 189 μg.h/g, respectively, which were much lower than those of ENR in the same tissue (Table 2). The T_1/2λ*z*_ values of CIP in the plasma, muscle/skin, kidney, and liver were 46.7 h, 127 h, 103 h, and 129 h, respectively. Therefore, it can be seen that CIP was eliminated faster in the plasma and kidney than ENR, while the other tested tissues were the opposite.

**Table 1:** Pharmacokinetic parameters of enrofloxacin in the plasma and tissues of crucian carp after oral administration of a single dose (20 mg/kg b.w.) at 28°C.

**Parameters**	**Unit**	**Plasma**	**Muscle/skin**	**Liver**	**Kidney**
T_max_	h	3.0	6.0	1.0	3.0
C_max_	ug/g(or mL)	8.93	13.9	31.2	27.3
T_1/2λ*z*_	h	67.4	82.8	94.4	114.4
Vd/F	L/kg	3.36	/	/	/
CL/F	L/h/kg	0.04	/	/	/
AUC_0−∞_	μg·h/mL	579	1,059	2,131	1,712
MRT	h	86.5	116	127	127

*T_max_ and C_max_ are the maximum concentration and the time to the maximum concentration, respectively; T_1/2λz_, elimination half-life; Vd/F, volume of distribution corrected for bioavailability; CL/F, body clearance corrected for bioavailability; AUC_0−∞_, the area under the concentration-time curve from zero to infinity; MRT, average residence time from zero to infinity*.

**Table 2:** Pharmacokinetic parameters of ciprofloxacin in the plasma and tissues of crucian carp after oral administration of a single dose (20 mg/kg b.w.) of enrofloxacin at 28°C.

**Parameters**	**Unit**	**Plasma**	**Muscle/skin**	**Liver**	**Kidney**
T_max_	h	3.0	6.0	24.0	12.0
C_max_	ug/g(or mL)	0.19	0.48	1.85	0.97
T_1/2λ*z*_	h	46.7	127	129	103
AUC_0−∞_	μg·h/mL	10.5	48.6	554	189
MRT	h	65.1	174	230	184

*T_max_ and C_max_ are the maximum concentration and the time to the maximum concentration, respectively; T_1/2λz_, elimination half-life; Vd/F, volume of distribution corrected for bioavailability; CL/F, body clearance corrected for bioavailability; AUC_0−∞_, the area under the concentration-time curve from zero to infinity; MRT, average residence time from zero to infinity*.

### Pharmacokinetics of ENR and CIP in the Multiple-Dose Oral Administration

**Figure 4:**
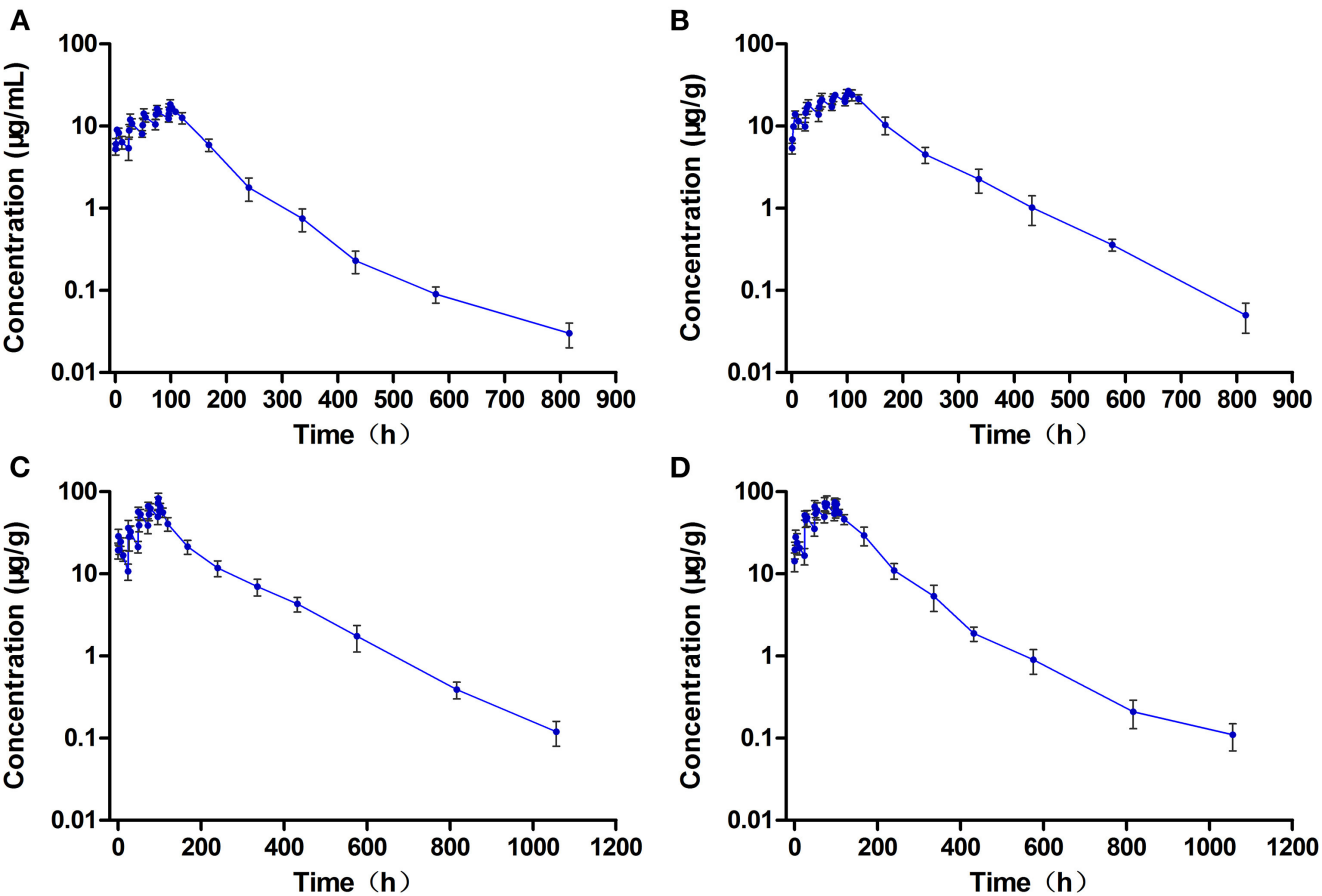
The concentration vs. time curves of enrofloxacin in the plasma **(A)**, muscle/skin **(B)**, liver **(C)**, and kidney **(D)** of crucian carp during and after multiple oral doses of enrofloxacin (20 mg/kg b.w. daily for 5 days) at 28°C (*n* = 6).

**Figure 5:**
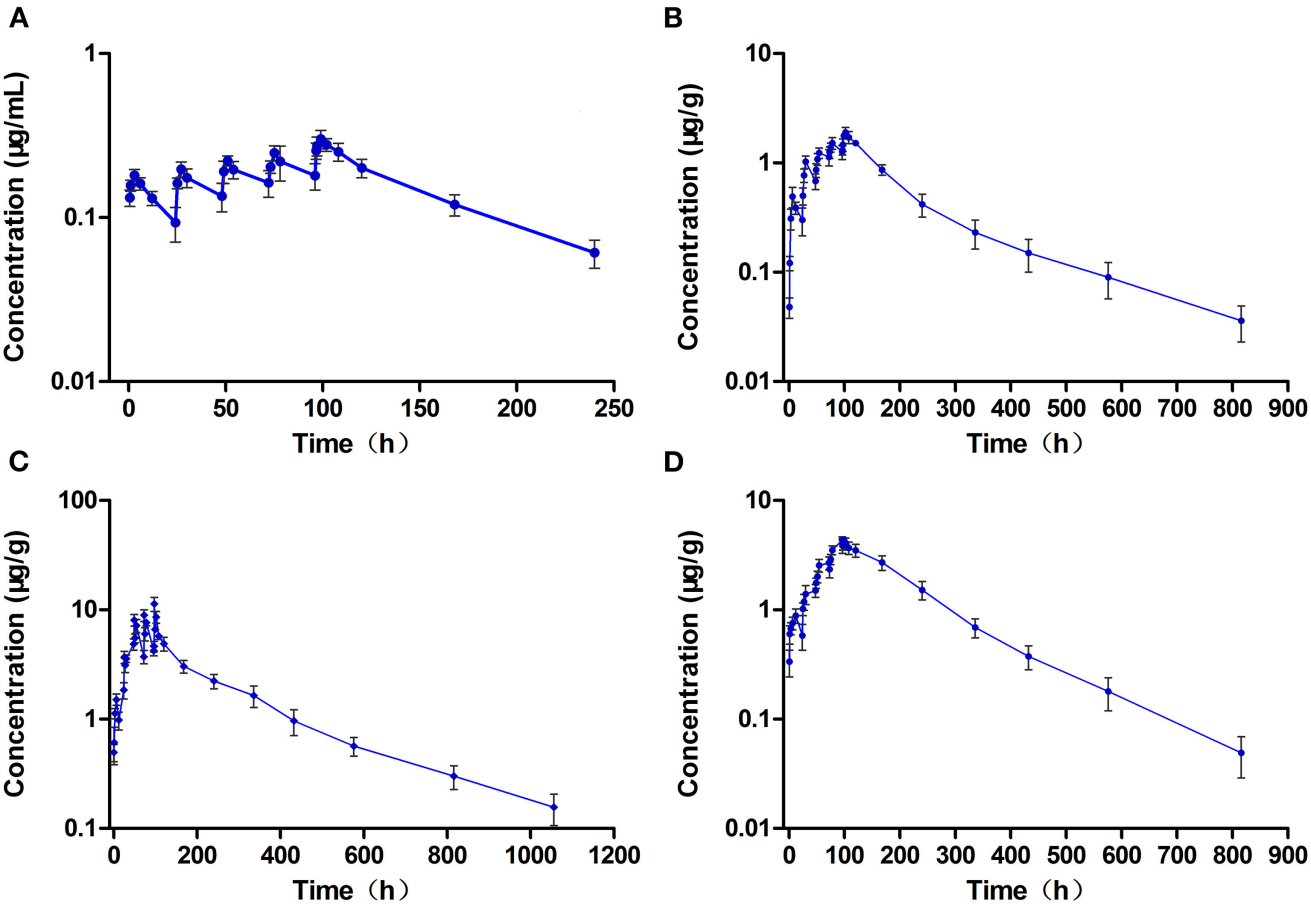
The concentration vs. time curves of ciprofloxacin in the plasma **(A)**, muscle/skin **(B)**, liver **(C)**, and kidney **(D)** of crucian carp during and after multiple oral doses of enrofloxacin (20 mg/kg b.w. daily for 5 days) at 28°C (*n* = 6).

The levels of ENR and CIP observed in the plasma and tested tissues of crucian carp during and after the multiple-dose oral administration are shown in Figures 4A–D, 5A–D, respectively. In the multiple-dose study, ENR exhibited two peaks in the kidney at 1 h and 6 h after the second dose, a new finding has not been reported before. Both the peak and trough concentrations of ENR and CIP in the plasma and tissue samples presented an increasing trend with an increasing number of doses. The maximum concentrations of ENR in the plasma, muscle/skin, liver, and kidney were 18.4 μg/ml, 26.8 μg/g, 82.8μg/g, and 74.5 μg/g, obtained at 3, 6, 1, and 1 h after the last dose, respectively (Table 3). These values were 2.04, 1.92, 2.89, and 2.65 times higher than the peak concentrations after the first dose in the corresponding plasma and tissues, respectively, showing significant accumulation of ENR in the crucian carp.

**Table 3:** Pharmacokinetic parameters of enrofloxacin in the plasma and tissues of crucian carp after oral administration of multiple doses (20 mg/kg b.w., for five consecutive doses, with an interval of 24 h between each dose) at 28°C (Time from the first dose).

**Parameters**	**Unit**	**Plasma**	**Muscle/skin**	**Liver**	**Kidney**
T_max_	h	99.0	102	97.0	97.0
C_max_	ug/g(or mL)	18.4	26.8	82.8	74.5
T_1/2λ*z*_	h	76.4	91.5	114	148
AUC_0−∞_	μg·h/mL	1,291	2,508	6,238	6,188
AUC_ss_	μg·h/mL	362	570	1,340	1,393
C_trough_	ug/g(or mL)	12.4	19.8	40.6	46.1
Cav	ug/g(or mL)	15.1	23.7	55.8	58.1
DF	-	0.40	0.30	0.76	0.49
MRT	h	72.9	93.3	98.3	93.5

*The expression of T_max_, C_max_, T_1/2λz_, AUC_0−∞_, MRT are consistent with those in Table 1. AUC_ss_, steady-state area under the concentration-time curve; C_trough_, the concentration at the end of the dosing interval at steady state; C_av_, the concentration of average steady-state drug; DF, the fluctuatation of drug concentration*.

The T_1/2λ*z*_ values of ENR in the plasma, muscle/skin, liver, and kidney were estimated as 76.4 h, 91.5 h, 114 h, and 148 h, respectively, which were somewhat longer than those after a single dose. The estimated AUC_0−∞_ values of ENR in the liver, kidney, muscle/skin, and plasma were 6,238 μg.h/g, 6,188 μg.h/g, 2,508 μg.h/g, and 1,291 μg.h/ml, respectively, and followed the order as liver > kidney > muscle/skin > plasma, which was consistent with that after a single dose, showing that ENR was most distributed in the liver.

The C_max_ of CIP in the plasma, muscle/skin, liver, and kidney was 0.30 μg/ml, 1.91 μg/g, 11.3 μg/g, and 4.41 μg/g, respectively, and they were shown at 3 h, 6 h, 1 h, and 3 h after the end of treatment (Table 4).CIP, like ENR, also showed a certain degree of accumulation in all the tested tissues and plasma, and the accumulation of CIP in the kidney was very evident, far more than ENF. The values of AUC_0−∞_ of CIP in the plasma and tissues also followed the same order of liver > kidney > muscle/skin > plasma, showing that CIP was also most distributed in the liver.

**Table 4:** Pharmacokinetic parameters of ciprofloxacin in the plasma and tissues of crucian carp after oral administration of multiple doses (20 mg/kg b.w., for five consecutive doses, with an interval of 24 h between each dose) at 28°C (Time from the first dose).

**Parameters**	**Unit**	**Plasma**	**Muscle/skin**	**Liver**	**Kidney**
T_max_	h	99.0	102	97.0	99.0
C_max_	ug/g(or mL)	0.30	1.91	11.3	4.41
T_1/2λ*z*_	h	70.1	107	175	110
AUC_0−∞_	μg·h/mL	26.3	227	1,130	623
AUC_ss_	μg·h/mL	5.99	40.4	154	91.6
C_trough_	ug/g(or mL)	0.18	1.26	4.24	3.50
Cav	ug/g(or mL)	0.25	1.68	6.40	3.82
DF	-	0.48	0.38	0.11	0.24
MRT	h	92.5	122	163	151

*The expression of T_max_, C_max_, T_1/2λz_, AUC_0−∞_, MRT are consistent with those in Table 1. AUC_ss_, steady-state area under the concentration-time curve; C_trough_, the concentration at the end of the dosing interval at steady state; C_av_, the concentration of average steady-state drug; DF, the fluctuatation of drug concentration*.

### Withdrawal Period Data

**Figure 6:**
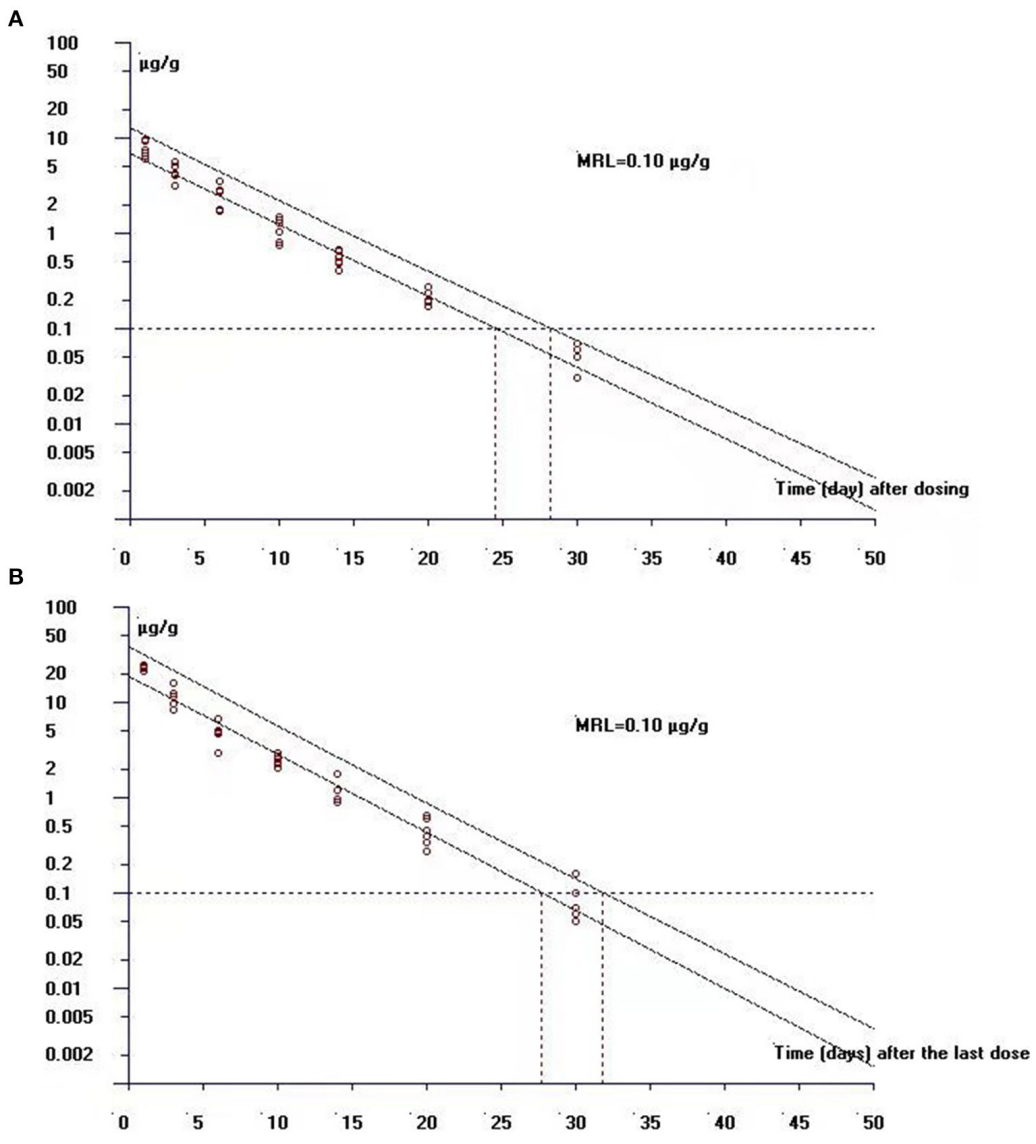
The theoretical withdrawal periods of enrofloxacin in crucian carp after **(A)** single (20 mg/kg b.w.) and **(B)** multiple dose (20 mg/kg b.w. daily for 5 days) oral administration at 28°C.

In the single-dose group, the concentrations of ENR and CIP in the edible tissue of muscle/skin below the LOQ (0.03 μg/g) can be achieved at 40 days and 20 days after the dosing, respectively, while in the multiple-dose group, both of the ENR and CIP concentrations were undetectable at 40 days after completion of the treatment regimen. The log-transformed concentration-time data of muscle plus skin were subject to a linear regression analysis, and the withdrawal period was estimated as the time when the upper one-sided 95% tolerance limit was below the established MRL of 0.1 μg/g with 95% confidence. The withdrawal periods were determined as 28.3 days and 31.9 days (rounded to 29 days and 32 days) in crucian carp held at 28°C after single and multiple oral gavage administration, respectively (Figures 6A,B).

## Discussion

In this study, the pharmacokinetics and residue elimination rules of ENR (20 mg/kg b.w.) and its metabolite CIP were investigated in crucian carp after single and multiple oral doses (five consecutive administrations with an interval of 24 h each time). In the single-dose group, the T_max_ of ENR in the plasma, muscle/skin, liver, and kidney were achieved at 3.0 h, 6.0 h, 1.0 h, and 3.0 h, independently, and the T_max_ values were similar to those in the plasma and muscle of snakehead (19), showing a slow absorption. This phenomenon was also found in juvenile Atlantic salmon (27), seabass (11), and brown trout (8), for which the T_max_ values were 2.87, 8, and 8 h in the plasma, independently. However, the present T_max_ value of 3 h in plasma was much longer than previously reported in crucian carp (T_max_ was 0.5 h for the plasma) (5) after the same oral dose of ENR solution without food, indicating that food has a great influence on the absorption of ENR in the crucian carp (28). In addition, two ENR peaks were observed in the liver and kidney after each dose administration (since the second dose). Double peaks coupled with the long half-lives suggest the possibility of hepato-enteric circulation (5).

In the present study, in both dose groups, the T_1/2λ*z*_ values of ENR in the plasma and tested tissues followed the order of kidney > liver > muscle/skin > plasma, indicating that the ENR clearance was slowest from kidney and fastest from plasma. In addition, we found that the T_1/2λ*z*_ values of ENR in the plasma and all the tested tissues in the multi-dose group were somewhat longer than those in the single-dose group. For some drugs, increased doses may result in a deviation from the pharmacokinetic profile of a single dose, and an increase in the elimination half-life is usually attributed to the saturation of enzyme system (29). After multiple doses, a significant accumulation of ENR was observed in the plasma and all the tested tissues of crucian carp. It is generally believed that when the drug dosing interval was equivalent to the elimination half-life, steady-state concentration would be achieved by 5–6 repeat doses (22). However, the present results showed that ENR was very slowly eliminated from the plasma and tissues of crucian carp, the calculated T_1/2λ*z*_ values of ENR in the plasma, muscle/skin, liver, and kidney of crucian carp after repeat dose were 76.4, 91.5, 114, and 148 h, respectively, which were significantly longer than the dosing interval of 24 h. Therefore, ENR is very likely to accumulate in crucian carp. This phenomenon has also been observed in the northern snakehead (19) and the Pacific white shrimp (22) after a repeat dose. As ENR exhibited a significant accumulation in the crucian carp during multiple-dose administration, reducing the dose, increasing the dosing interval or both should be considered while developing a dosage regimen.

In the single-dose group, the apparent volume of distribution (Vd/F) was estimated to be 3.36 L/Kg, showing that ENR was widely distributed in the crucian carp. In both dose groups, the AUC_0−∞_ for both ENR and CIP followed the order of liver > kidney > muscle/skin > plasma. A similar order was found in the tissue distribution of ENR and CIP in the northern snakehead (19) and ENR in the grass carp (12), suggesting that ENR and its metabolite CIP were mostly distributed in the liver, and the liver may play an important role in the metabolism of ENR.

In this study, CIP could be detected in the plasma and all the tested tissues of crucian carp after an oral dose of ENR. However, the levels of CIP were extremely lower than those of ENR, and the AUC ratios of CIP and ENR in the plasma of crucian carp were calculated as 1.81 and 2.04% in the single-dose and multiple-dose groups, respectively. The results obtained were similar to those previously observed in the northern snakehead (19), striped catfish (30), and red pacu (31), for which the AUC ratios of CIP and ENR in the plasma were in the range 1.84**–**5.90%. However, CIP was not detected in brown trout (8), showing that the conversion rate of ENR in aquatic animals was very low, and most of them existed in the form of the original drug. After repeated administrations, CIP was accumulated in various tissues, and it was more obvious in the kidney, indicating that the kidney is likely the primary excretory organ of CIP. As the number of administration increased, the metabolic rate of ENR was greater than the elimination rate of CIP.

Enrofloxacin MICs against bacterial isolates from aquatic animals were previously reported to be 0.06–0.4 μg/ml for *Aeromonas hydrophila*, 0.005–1.28 μg/ml for *Aeromonas salmonicida*, 0.005–0.16 μg/ml for *Vibrio salmonicida*, and 0.005–0.03 μg/ml for *Yersinia ruckeni* (6, 9). However, the values quoted here are very old and may no longer be valid now. Fluoroquinolone antimicrobial agents are identified to display concentration-dependent bactericidal activity, and the pharmacodynamics predictors of AUC_24h_/MIC ratios ≥ 125 and C_max_/MIC ratios ≥ 10 have been reported to provide clinical and bacteriological success and to prevent the emergence of resistance (32). In the present study, assuming that the MIC was 1.84 μg/ml, the ratios of C_max_/MIC and AUC_ss_/MIC in the plasma of crucian carp after repeat dose were calculated as 10.01 and 196.6, respectively, those values were just higher than ratios of 10 for C_max_/MIC and 125 for AUC_24h_/MIC, respectively. Therefore, it can be seen that our ENR dosing regimen will have a very good effect on pathogens with a MIC value of <1.84 μg/ml. However, fluoroquinolones are on the WHO list of drugs that should be reserved for human use. Therefore, they should be used with caution in food animal production in order to reduce the drug use, thus minimizing the development of resistance.

Results showed that a withdrawal period of at least 29 days or 32 days was necessary for crucian carp to guarantee food security after single or multiple oral gavage administration of ENR. In the whole animal experiments, the temperature was always kept at 28°C, therefore, the withdrawal periods were further calculated as 812 degree days and 896 degree days after single and multiple oral gavage administration of ENR, respectively. The pharmacokinetics of a drug in the fish can be affected by many factors, such as fish species, dosing regimen, route of administration, and water temperature, therefore, those factors should be carefully considered to set the clinical withdrawal period.

## Conclusion

The present results showed that ENR had a wide distribution in the tissues of the crucian carp, but the elimination was very slow. ENR and CIP exhibited large distribution in the liver, indicating that the liver was an important organ for metabolism. Based on the calculated PK/PD indices of C_max_/MIC and AUC_24h_/MIC, the multiple-dosing regimen would have a very good effect on pathogens with a MIC value of <1.84 μg/ml. The residue depletion studies indicated that a withdrawal period of at least 812 or 896 degree days was necessary for crucian carp to guarantee food security after single or multiple oral gavage administration of ENR.

## Data Availability Statement

The original contributions presented in the study are included in the article/Supplementary Material, further inquiries can be directed to the corresponding authors.

## Ethics Statement

The animal study was reviewed and approved by Laboratory Animal Ethics Committee of Pearl River Fisheries Research Institute, CAFS.

## Author Contributions

QS, YY, and JW designed the project. QS, HH, JW, WX, LM, HZ, LL, and SL conducted the animal experiment and collected samples. QS, YY, and HH extracted and collected all the concentration-time data. QS performed the data analysis and wrote the manuscript. GZ and XZ provided support to conduct the work with the crucian carp. All authors contributed to the article and approved the submitted version.

## Funding

This work was funded by the National Key Research and Development Program of China (project No. 2017YFC1600704), the China-ASEAN Maritime Cooperation Fund (project No. CAMC-2018F), and the Guangzhou Science and Technology Plan Project (project No. 202102020335).

## Conflict of Interest

The authors declare that the research was conducted in the absence of any commercial or financial relationships that could be construed as a potential conflict of interest.

## Publisher's Note

All claims expressed in this article are solely those of the authors and do not necessarily represent those of their affiliated organizations, or those of the publisher, the editors and the reviewers. Any product that may be evaluated in this article, or claim that may be made by its manufacturer, is not guaranteed or endorsed by the publisher.
